# 2:1 ketogenic diet and low-glycemic-index diet for the treatment of chronic and episodic migraine: a single-center real-life retrospective study

**DOI:** 10.1186/s10194-023-01635-9

**Published:** 2023-07-28

**Authors:** Yan Tereshko, Simone Dal Bello, Cherubino Di Lorenzo, Sara Pez, Alice Pittino, Roberto Sartor, Francesca Filippi, Christian Lettieri, Enrico Belgrado, Riccardo Garbo, Giovanni Merlino, Gian Luigi Gigli, Mariarosaria Valente

**Affiliations:** 1 Clinical Neurology Unit, Udine University Hospital, Piazzale Santa Maria della Misericordia 15, Udine, 33100 Italy; 2 Department of Medico-Surgical sciences and Biotechnologies, Sapienza University of Rome Polo Pontino, Latina, 04100 Italy; 3 Neurology Unit, Udine University Hospital, Piazzale Santa Maria della Misericordia 15, Udine, 33100 Italy; 4 Neurology Unit, Hospital of Gorizia, Gorizia, 34170 Italy; 5 Department of Medicine (DAME), University of Udine, Via Colugna 50, Udine, 33100 Italy

**Keywords:** Ketogenic diet, Migraine, Headache, Migraine prophylaxis, Low-glycemic index diet

## Abstract

**Aims:**

The evidence supporting the efficacy of dietary preventive therapy in migraine is rising, particularly regarding the ketogenic diet. However, less evidence exists for the Low-Glycemic Index Diet and the 2:1 KD. This retrospective single-center real-life study aims to evaluate the efficacy of a 2:1 ketogenic diet and a Low-Glycemic-index Diet in chronic and high-frequency episodic migraine.

**Methods:**

Sixty patients with high-frequency episodic and chronic migraine were treated with either a Low-Glycemic-index diet (39 patients) or a 2:1 (21 patients) ketogenic diet for three months. We collected data on the migraine frequency and intensity and the MIDAS and HIT-6 scores through the headache diary. Anthropometric measurements (BMI, fat mass, free fat mass, and weight) were also collected and analyzed similarly. Data obtained at the baseline and after three months of each diet were compared.

**Results:**

Migraine intensity, frequency, MIDAS and HIT-6 scores, fat mass, weight, and BMI improved in both diet groups.

**Conclusions:**

Both diets are effective in reducing migraine symptoms and migraine-related disability.

## Introduction

Migraine is one of the most frequent neurological diseases. It mainly affects patients in their most productive years. It can cause very high disability and poor quality of life, up to being classified as the second leading cause of disability worldwide [1]. There is a strong need in clinical practice to use practical approaches in the preventive treatment of migraine to reduce the burden of this disease. Although new preventive therapies for migraine, the Calcitonin Gene-Related Peptide monoclonal antibodies (CGRP mAbs), have proven to be very effective, some patients did not respond optimally; this is the case of overweight patients with migraine [2].

For this reason, non-pharmacological preventive therapies are rising these years, offering a promising approach even in combination with conventional pharmacotherapies [3, 4]. Among the non-pharmacological preventive treatments for migraine, nutritional interventions have been studied to offer innovative therapeutic strategies for primary headaches to avoid adverse events and drug interactions [5–7]. Moreover, there is strong evidence that the Ketogenic Diet (KD) has important therapeutic implications in several neurological diseases, influencing neuronal plasticity and exerting neuroprotective effects [8]. KD has been tested with significant efficacy in treating migraine [9]. Although its mechanism of action is not yet fully elucidated, it improves brain metabolism and excitability, mitigating neuro-inflammation through the production of ketone bodies [10]. To date, KD therapy has been successfully applied in drug-resistant migraine and cluster headache [11–14]; these studies have prompted the experts in nutrition and headache to publish the clinical recommendation for the application of KD in patients with headache [15]. Several ketogenic diets are proposed for the treatment of migraine, and they have in common the restriction of carbohydrates and an adequate protein intake, differentiating according to the variation of the lipid-to-carbohydrate and protein ratio. These include the low-glycemic-index diet (LGID) [16], the low-calorie-ketogenic-diet (LCKD) [17], the very-low-calorie-ketogenic-diet (VLCKD) [11, 12, 17, 18], the Modified Atkins Diet (MAD) [18–21], and the Classic KD [17, 19]. Classic KD is a low carbohydrate diet based on a drastic reduction in carbohydrate intake, associated with a relative increase in the proportion of fats and regular intake of proteins. During KD, without exogenous glucose as an energy source, fatty acids are mobilized from fat deposits and transported to the liver for conversion into ketone bodies (acetoacetate, β-hydroxybutyric acid, and acetone). The ketone bodies are then distributed to metabolically active tissues, namely skeletal muscle, brain, and heart, representing an essential energy source as they are converted into acetyl coenzyme A (acetyl-CoA), the substrate of the Krebs cycle. Experts suggest a 3:1 or 4:1 KD ratio in treating migraine as a preventive therapy, while the 2:1 KD ratio is less studied in this setting [15].

The LGID is characterized by a high daily intake of fats (60%), a low intake of carbohydrates (40-60 g) with a glycemic index < 50 (low tendency to increase to elevate blood glucose), and an intake of proteins of about 30%; the LGID has proven to be effective in epileptic patients that are unable to undergo KD due to scarce tolerability or palatability. LGID exerts its actions through the induction of the production of ketone bodies, although less pronounced than classic KD, and the prevention of dramatic fluctuations in blood glucose. There is limited data regarding the use of LGID in the migraine setting in the literature [16, 22]. Still, its application is encouraged in migraineurs that present scarce tolerability to classical KD [15]. The present study aims to retrospectively investigate the efficacy and impact on the quality of life of two different diet protocols (LGID and 2:1 KD) in patients with high-frequency episodic and chronic migraine, assessed with validated migraine-specific questionnaires such as Migraine Disability Assessment (MIDAS) and Headache Impact Test (HIT-6); moreover. Moreover, we evaluated the adverse events and the effect on anthropometric features of the two diets.

## Methods

### Study design, participants, and eligibility

This is a retrospective analysis of prospectively collected data. We retrospectively collected data from 87 patients with a clinical diagnosis of migraine (both chronic or high-frequency episodic), treated with a 2:1 KD or Low-Glycemic-index diet as a preventive therapy for migraine from January 2020 to July 2022 in our nutritional outpatient clinic (Clinical Neurology Unit, Ospedale S. Maria della Misericordia, Udine). High-frequency episodic migraineurs were patients with 8–14 days of migraine per month, while chronic migraineurs had 15 or more days of migraine per month [23]. Patients were recruited from those proposed by a headache clinician; they were interested in reducing their weight, fat mass, and migraine disability but were reluctant to try new pharmacological preventive therapies. The assignment to each regimen had been decided based on the patient’s BMI: overweight patients (BMI between 25 and 29.9 Kg/m^2^) were assigned to LGID due to its lower fat content, higher palatability, and tolerability; this diet regimen could improve the adherence and reduce the dropouts since dieting in overweight patients is more challenging [24]. Patients with a BMI of 18.5–24.9 Kg/m^2^ were given a 2:1 KD; among the ketogenic diets, this protocol is the most palatable and tolerable, although less than the LGID. Concomitant anti-migraine prophylaxis was permitted if it was present for at least three months before the diet and was not discontinued or its posology was not modified. Seventeen patients stopped the diet before reaching the three-month evaluation (ten patients in the 2:1 KD group and seven in the LGID group); this was due to intolerance to the diet carbohydrate restriction (seven patients in the 2:1 KD group and two in the LGID group), excessive loss of time in the meal preparation and inability to consume these meals at work (two patients in the 2:1 KD group), poor tolerance to the diet (persistent abdominal pain in one patient in the 2:1 KD group after one week of dieting). Four patients of the LGID group dropped out after a few days (3–4 days) due to loss of confidence in the diet; one patient in the LGID group dropped out due to unknown reasons; the data of ten patients (four in the LGID group and six in the 2:1 KD group) were missing.

**Fig. 1:**
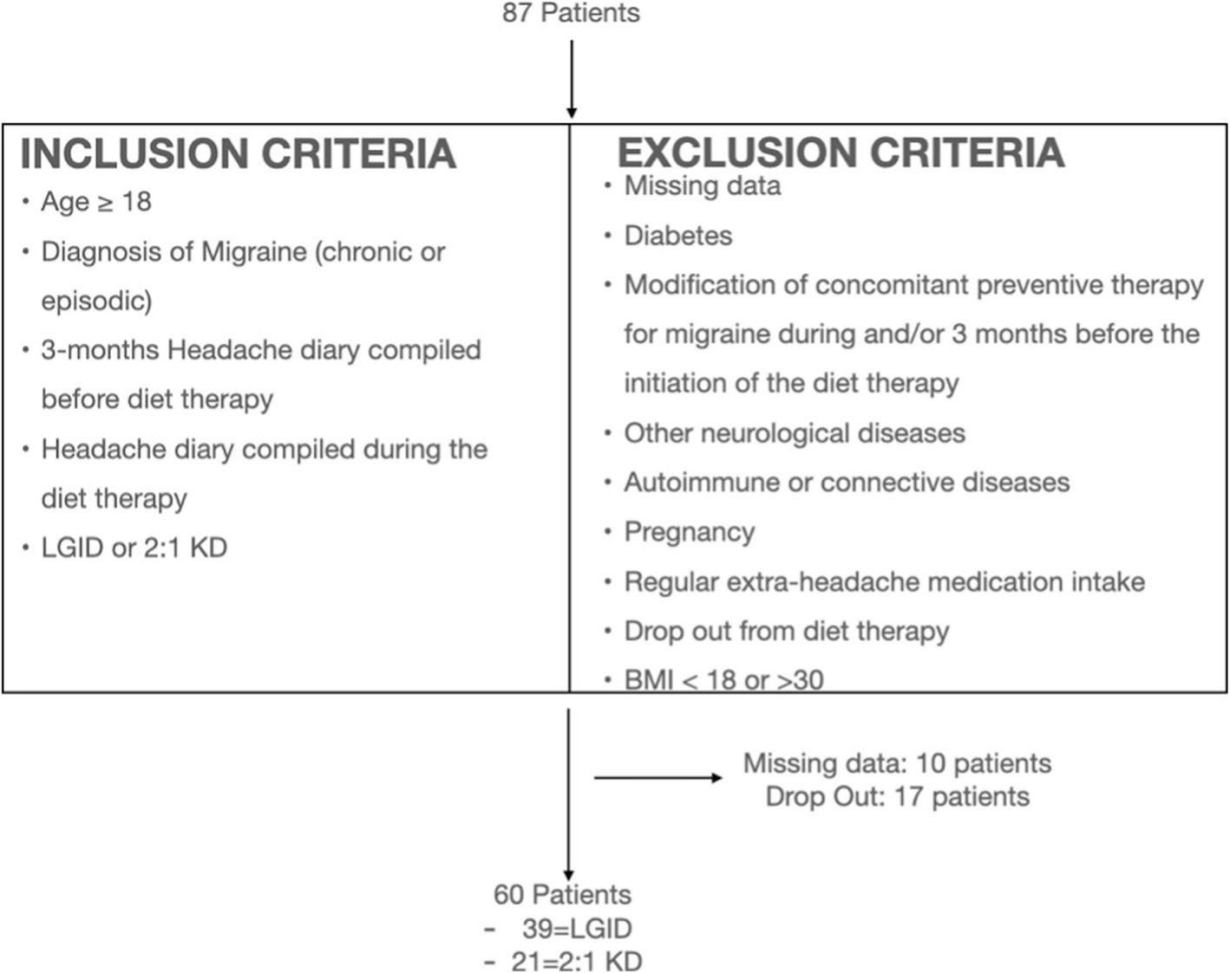
Shows the inclusion and exclusion criteria in our study

Sixty patients were considered suitable for statistical analysis due to exclusion criteria. The flowchart, with the inclusion and exclusion criteria, is shown in Fig. 1. All the patients underwent baseline nutritional and neurological examinations. We collected data concerning demographics, migraine features, previous migraine preventive therapies, and comorbidities; in the baseline evaluation, we also collected data regarding migraine intensity, frequency, MIDAS, and HIT-6. Migraine intensity and frequency were assessed using a headache diary filled at least one month before each diet and during treatment. In the basal nutritional evaluation, the patients were thoroughly studied with Bioelectrical Impedance Analysis (BIA) 101 BIVA PRO (Akern®) to determine the Fat Mass (FM), the Free-Fat Mass (FFM), the intracellular (ICW) and extracellular water (ECW); data regarding height, weight, Body Mass Index (BMI), and dietary preferences were also collected. A 1-month evaluation with only the nutritionist was performed to assess the adherence to the diet with the aid of a diet diary. After three months of diet, patients were reassessed from a neurological and nutritional point of view.

### Ethical aspects

The study was conducted in accordance with the Declaration of Helsinki and approved by the Institutional Review Board of the University of Udine IRB-DAME (Prot IRB: 103/2022, approved on 11 July 2022). All the patients gave formal written consent for nutritional treatment with the ketogenic diet and for their clinical data to be used for scientifical purposes.

### Ketogenic diet therapy

Our nutritionist counseled patients to develop and personalize their diet; the content of proteins and fats was tailored based on the anthropometric measurements, BIA, and level of daily physical activity. A Low-glycemic index diet was prescribed for patients with a 25-29.9 Kg/m2 BMI due to its higher palatability, tolerability, and lower fat content. The protein content was calculated based on the lean mass obtained with the BIA; the conversion in grams was performed considering the patient’s activity level and the ideal weight. The quantity of carbohydrates was fixed at 30 g per day and not liberalized. In our case, the amount of fats was equal to the sum of carbohydrates and proteins, obtaining a ratio of 1:1, and the total calories of the diet were 1300–1500 calories a day (Kcal/day). A 2:1 KD was prescribed for a BMI of 18.5–24.9 Kg/m^2^. The protein content was calculated as we did for the LGID; the number of carbohydrates was fixed at 30 g daily. In this dietary regimen, the number of fats equals double the sum of carbohydrates and proteins (2:1 ratio). The total calories of this diet were 1600–2300 Kcal/day.

### Endpoints

#### Primary endpoint

The study’s primary endpoint was to assess the efficacy of the two diets based on migraine intensity, frequency, and MIDAS and HIT-6 scales. For this purpose, we compared the migraine frequency and intensity of the patients at the baseline with the data obtained in the last month of the dietary regimen; similarly, we compared the MIDAS and HIT-6 scales. Both high-frequency episodic and chronic migraineurs in each group were studied similarly.

#### Secondary endpoint

To compare the effect of the two diet protocols (LGID and 2:1 KD) on the anthropometric features (weight, BMI, FM, FFM, ICW, ECW, and phase angle). The same statistical analysis was performed in both chronic and high-frequency episodic migraineurs.

### Data and statistical analysis

Since this is a retrospective study, the population we analyzed was based on formal statistics but on the availability of overweight and normoweight patients interested in undergoing dietary treatment for 3–6 months instead of standard preventive therapies for migraine. However, based on our previous analysis [14], we calculated a power of the study of 0.80 for the 2:1 KD group (Effect size 0.64; alpha error 0.05; two-tails); for the LGID group, since there are not sufficient data in the literature, we set the effect size to 0.5 (alpha error 0.05; two tails) and obtained a power of 0.86. A descriptive analysis of the study population’s main features was performed using mean ± SD for continuous variables and absolute and relative frequencies for categorical variables. A Shapiro-Wilk test was used to assess the normal distribution of data. Comparisons were performed as appropriate using a t-test or Mann-Whitney’s test. We employed the Chi-square test to compare qualitative variables. A paired t-test or a Wilcoxon test was used to compare the clinical data at the baseline and after three months of each diet. All analyses used Stata/SE (version 15.1, StataCorp) for Mac OS. All 2-tailed statistical significance levels were set at *p* < 0.05.

## Results

Thirty-nine patients (31 females and eight males) were treated with a Low-Glycemic-index diet, while twenty-one (17 females and four males) were treated with a 2:1 ketogenic diet. Most of our population was composed of female patients (80%), in agreement with the migraine prevalence data in the literature [25]. Twenty-three patients in the LGID group presented chronic migraine, while 16 had high-frequency episodic migraine; in the 2:1 KD group, ten patients out of 21 had chronic migraine, and the remaining had high-frequency episodic migraine.

Detailed characteristics of the two groups are described in Table 1.

**Table 1:** Shows the two diet groups’ demographics, main comorbidities, anthropometric features, and migraine characteristics

Variable	LGID (mean ± SD or N°,/%)	2:1 KD (mean ± SD or N°,/%)
Number of patients	39	21
Age	46.333 ± 14.582	44.143 ± 14.698
Female Sex	31/39 (79.48%)	17/21(80.95%)
BMI	26.731 ± 5.331	21.814 ± 2.681
Weight (Kg)	74.756 ± 15.013	63.819 ± 12.640
FM (Fat Mass)	24.197 ± 10.465	15.900 ± 6.197
Fat-Free Mass (FFM)	51.121 ± 8.970	47.762 ± 9.535
Phase Angle	5.497 ± 0.755	5.338 ± 0.503
Intracellular Water (ICW)	19.666 ± 4.204	17.833 ± 4.716
Extracelular Water (ECW)	18.142 ± 3.052	17.248 ± 3.219
Years of Migraine	22.821 ± 17.916	25.524 ± 17.043
Smoking	6/39(15.38%)	5/21(23.81%)
Previous Migraine prophylaxis	2.308 ± 2.015	2.619 ± 2.247
Concomitant prophylaxys	19/39(48.72%)	14/21(66.67%)
Symptomatic intake/month	15.194 ± 10.765	14.048 ± 17.348
Chronic Migraine	23/39 (58.97%)	10/21 (47.62%)
**Migraine Characteristics**		
MIDAS	75.821 ± 80.493	69.726 ± 58.665
HIT-6	64.385 ± 7.697	66.476 ± 5.819
Migraine days/month frequency	18.744 ± 8.478	15.875 ± 8.374
Migraine Intensity (NRS)	8.051 ± 1.075	8.143 ± 0.793
Familiarity for migraine	12/39(30.77%)	10/21(47.62%)
Throbbing quality of pain	25/39(64.10%)	15/21(71.43%)
Migraine with Aura	8/39(20.51%)	6/21(28.57%)
Photophobia	32/39(82.05%)	19/21(90.48%)
Phonophobia	28/39(71.79%)	18/21(85.71%)
Osmophobia	7/39(17.95%)	4/21(19.05%)
Nausea	34/39(87.79%)	18/21(85.71%)
Vomit	15/39(38.46%)	10/21(47.62%)
**Comorbidities**		
Major Depressive Disorder	6/39 (15.38%)	1/21 (4.76%)
Concomitant MOH	18/39(46.15%)	5/21(23.81%)

Both groups presented a statistically significant reduction in migraine days/month and intensity; MIDAS and HIT-6 scales also improved significantly. Both chronic and high-frequency episodic migraineurs in each group had significant improvement in these variables. The detailed statistics are presented in Table 2.

**Table 2:** Shows both groups’ migraine days/month, intensity, HIT-6, and MIDAS at the baseline and after three months of each diet; results for chronic and high-frequency episodic migraineurs are also presented. There was a significant reduction in all variables studied. A paired t-test or a Wilcoxon test was used to compare; significance was determined with a *p*-value of 0.05

	Baseline (mean ± SD)	3 months (mean ± SD)	*p-*value
**LGID (*n* = 39)**			
MIDAS	75.821 ± 80.493	32.051 ± 52.307	**< 0.001**
HIT-6	63.905 ± 8.854	52.714 ± 11.538	**< 0.001**
Migraine days/month	18.744 ± 8.478	9.154 ± 10.676	**< 0.001**
Migraine Intensity (NRS)	8.051 ± 1.075	4.846 ± 2.852	**< 0.001**
**LGID chronic (*n* = 23) **			
MIDAS	84.174 ± 89.294	44.652 ± 64.558	**< 0.001**
HIT-6	64.783 ± 6.466	55.870 ± 12.871	**0.001**
Migraine days/month	24.435 ± 6.302	12.522 ± 12.616	**< 0.001**
Migraine Intensity (NRS)	7.739 ± 0.964	5.000 ± 2.908	**< 0.001**
**LGID episodic (*n* = 16) **			
MIDAS	63.813 ± 66.774	13.938 ± 15.004	**< 0.001**
HIT-6	63.813 ± 9.389	52.625 ± 12.842	**< 0.001**
Migraine days/month	10.563 ± 1.672	4.313 ± 3.591	**< 0.001**
Migraine Intensity (NRS)	8.500 ± 1.095	4.625 ± 2.849	**< 0.001**
**2:1 KD (*n* = 21) **			
MIDAS	69.762 ± 58.665	31.524 ± 52.018	**< 0.001**
HIT-6	61.231 ± 11.840	53.250 ± 16.341	**< 0.001**
Migraine days/month	15.857 ± 8.374	6.476 ± 5.776	**< 0.001**
Migraine Intensity (NRS)	8.143 ± 0.793	5.857 ± 2.744	**< 0.001**
**2:1 chronic (*n* = 10) **			
MIDAS	97.900 ± 73.847	52.500 ± 69.728	**0.015**
HIT-6	66.200 ± 6.844	54.700 ± 20.106	**0.022**
Migraine days/month	22.100 ± 8.386	8.800 ± 6.697	**0.013**
Migraine Intensity (NRS)	8.500 ± 0.707	6.000 ± 2.449	**0.002**
**2:1 episodic (*n* = 11) **			
MIDAS	44.182 ± 21.531	12.455 ± 13.995	**0.005**
HIT-6	66.727 ± 5.042	50.909 ± 12.169	**0.004**
Migraine days/month	10.182 ± 1.601	4.364 ± 4.007	**0.022**
Migraine Intensity (NRS)	7.818 ± 0.751	5.727 ± 3.101	**0.002**

After three months of therapy, the LGID and 2:1 ketogenic diet group had significantly reduced BMI, weight, and Fat Mass. The Fat-Free Mass was preserved regardless of the ketogenic ratio (see Table 3). Both chronic and high-frequency episodic migraineurs in each group presented the same results. The 2:1 KD group showed a significant increase in the phase angle; this significance was lost when the group was divided into chronic and high-frequency episodic migraine.

**Table 3:** Shows BMI, fat mass, free fat mass, phase angle, and intracellular and extracellular water at the baseline and after three months of each diet. The results for chronic and high-frequency migraineurs in each group are also presented. There was a significant reduction in BMI and fat mass in both groups. A paired t-test or a wilcoxon test was used to compare; significance was determined with a *p*-value of 0.05

	Baseline (mean ± SD)	3 months (mean ± SD)	*p-*value
**LGID (*n* = 39)**			
Weight (Kg)	74.756 ± 15.013	68.821 ± 13.516	**< 0.001**
BMI	26.731 ± 5.331	24.556 ± 4.704	**< 0.001**
FM	24.197 ± 10.465	18.113 ± 9.991	**< 0.001**
Phase Angle	5.497 ± 0.775	5.633 ± 0.722	0.123
FFM	51.121 ± 8.970	51.087 ± 8.663	0.667
H2O ICW	19.666 ± 4.204	19.950 ± 3.864	0.164
H2O ECW	18.142 ± 3.052	17.992 ± 3.219	0.528
**LGID chronic (*n* = 23) **			
Weight (Kg)	73.709 ± 13.042	67.596 ± 10.972	**< 0.001**
BMI	26.835 ± 3.782	24.516 ± 2.938	**< 0.001**
FM	24.200 ± 7.033	17.391 ± 5.959	**< 0.001**
Phase Angle	5.343 ± 0.732	5.457 ± 0.710	0.244
FFM	50.532 ± 8.801	50.532 ± 8.801	0.306
H2O ICW	19.509 ± 4.287	19.941 ± 3.902	0.073
H2O ECW	17.955 ± 3.565	17.964 ± 3.501	0.978
**LGID episodic (*n* = 16) **			
Weight (Kg)	76.263 ± 17.816	70.581 ± 16.753	**< 0.001**
BMI	26.581 ± 7.140	24.613 ± 6.586	**< 0.001**
FM	24.194 ± 14.315	18.863 ± 14.169	**< 0.001**
Phase Angle	5.719 ± 0.755	5.888 ± 0.682	0.211
FFM	52.056 ± 8.198	51.850 ± 8.694	0.770
H2O ICW	19.881 ± 4.217	19.962 ± 3.939	0.832
H2O ECW	18.400 ± 2.251	18.031 ± 2.898	0.244
**2:1 KD (*n* = 21) **			
Weight (Kg)	63.819 ± 12.640	59.819 ± 10.872	**< 0.001**
BMI	21.814 ± 2.681	20.386 ± 2.059	**< 0.001**
FM	15.900 ± 6.197	12.476 ± 4.589	**< 0.001**
Phase Angle	5.338 ± 0.503	5.633 ± 0.695	**0.041**
FFM	47.762 ± 9.535	47.067 ± 8.966	0.070
H2O ICW	17.833 ± 4.716	17.729 ± 4.444	0.856
H2O ECW	17.248 ± 3.219	16.814 ± 2.773	0.218
**2:1 chronic (*n* = 10) **			
Weight (Kg)	62.480 ± 11.683	58.310 ± 9.167	**0.010**
BMI	21.740 ± 2.409	20.350 ± 1.466	**0.009**
FM	15.110 ± 7.720	11.830 ± 5.383	**0.030**
Phase Angle	5.430 ± 0.600	5.850 ± 0.880	0.099
FFM	47.350 ± 10.419	46.480 ± 9.337	0.258
H2O ICW	17.620 ± 5.121	17.780 ± 5.218	0.767
H2O ECW	17.010 ± 3.464	16.340 ± 2.534	0.340
**2:1 episodic (*n* = 11) **			
Weight (Kg)	65.036 ± 13.902	61.191 ± 12.506	**0.002**
BMI	21.882 ± 3.023	20.418 ± 2.558	**< 0.001**
FM	16.618 ± 4.688	13.064 ± 3.904	**< 0.001**
Phase Angle	5.300 ± 0.560	5.436 ± 0.423	0.378
FFM	48.364 ± 9.660	47.600 ± 9.035	0.150
H2O ICW	18.027 ± 4.561	17.682 ± 3.871	0.376
H2O ECW	17.464 ± 3.134	17.245 ± 3.028	0.285

The LGID group reported constipation (12.82%), diarrhea (2.56%), abdominal pain (23.08%), and nausea (2.56%) as adverse events of the diet, while the 2:1 KD group reported constipation (23.81%) and abdominal pain (23.81%); there were no significant differences between the two groups (see Table 4). The symptoms were mild and occurred during the first month of the diet.

**Table 4:** Shows the patients’ adverse events during the three months of KD. There were no statistically significant differences between the two groups. A Chi-square test was used to compare the two groups; significance was determined with a *p*-value of 0.05

Side effect	LGID	2:1 KD	*p-*value
Constipation	5/39	5/21	0.276
Diarrhea	1/39	0/21	0.459
Abdominal pain	9/39	5/21	0.949
Nausea	1/39	0/21	0.459

Considering responders (≥ 50% reduction of migraine days) and not responders (< 50% reduction of migraine days) in both groups, the response rate was identical: 26/39 (66.67%) and 14/21 (66.67%) responders in the LGID group 2:1 KD group respectively.

## Discussion

In our study, LGID and 2:1 KD improved migraine intensity and frequency in high-frequency episodic and chronic migraineurs; both MIDAS and HIT-6 scales significantly improved. Efficacy data for KD and LGID are comparable with the literature [11, 14, 16, 17, 19], although the previous studies did not evaluate the HIT-6 and MIDAS scales. The efficacy of these diet regimes on migraine probably derives from a combination of factors: neurophysiological modulation with a secondary effect on cortical spreading depression, improvement of cerebral energy metabolism by acting on the mitochondrion, reduction of oxidative stress, anti-inflammatory effect, epigenetic modulation, improvement of the glutamate/GABA balance, modulation of ion channels, improvement of intracerebral glucose metabolism, correction of the metabolic syndrome, prevention of pronounced fluctuations in blood glucose, although the precise underlying mechanisms remain to be clarified [15]. Both dietary treatments reduced BMI, weight, and FM, as previously described for KD; this was true in chronic and high-frequency episodic migraine [11, 14]. ICW, ECW, and FFM did not modify significantly, according to the literature [14]. Overall, it is well known that improving migraine frequency and intensity is also linked to weight, FM, and BMI reduction, regardless of the type of diet used [11].

Moreover, the phase angle, a marker of inflammatory status [26], improved significantly in the 2:1 KD group; this could relate to a higher degree of ketone bodies production leading to a more pronounced anti-inflammatory activity. However, the significance of this finding is lost when considering the groups with chronic headaches and those with episodic high-frequency headaches, probably due to the low sample size. In our study, although the dietary protocols have proven to be effective in improving migraine three months after their application, it is impossible to determine the onset of their action in mitigating migraine symptoms. Interestingly, conflicting results emerge about the efficacy of KD therapy at one month of treatment: some studies, indeed, do not achieve satisfactory results when compared with other prophylactic therapies [16], while in others, KD appears to be effective already in the first month [11, 12, 18, 21]. The migraineurs examined have a clinically relevant burden from migraine, considering the number of pharmacological prophylaxes previously used, the number of symptomatic medications taken monthly, and the medication overuse headache (MOH) rate. The efficacy data collected on this population further corroborate the effectiveness of such dietary regimens on migraine. However, dietary intervention should be considered differently than pharmacological intervention due to a different mechanism of action, especially considering the new therapies directed against CGRP. However, the evidence shows that the nutritional approach can be a valuable weapon against migraine as a single therapy or combined with currently available pharmacological prophylactic therapies.

KD 2:1 and LGID diets are safe with usually minor adverse effects. The side effects found in our patient cohort aligned with the literature data [15]. The number of diet dropouts in our study was 22% (17/77 patients), comparable to that found in the literature [14].

### Limitations of the study

Our study has some limitations. First of all, the uncontrolled retrospective design of the study and the study sample were relatively small. Moreover, we did not assess ketonemia and ketonuria in our patients to determine whether adherence to the dietary regimen was adequate. Furthermore, both diets tend to induce a low ketone bodies production, and their detection is unnecessary; the improvement in both migraine and anthropometric features confirms the adherence to both dietary regimens. Finally, the sample is representative of only some of the population of migraineurs since it is composed by overweight and normoweight patients who were interested in undergoing dietary treatment for 3–6 months instead of standard migraine preventive therapies.

## Conclusions

Both LGID and 2:1 Ketogenic Diet therapy effectively reduced migraine symptoms and migraine-related disability. Each diet reduced weight and BMI, primarily due to fat mass loss. Further studies with larger samples and prospective design are necessary to confirm our results.

## Data Availability

The datasets used and/or analysed during this study are available from the corresponding author on reasonable request from any qualified investigator.

## Abbreviations

KD: Ketogenic diet
LGID: Low-glycemic index diet
VLCKD: Very low-calorie ketogenic diet
LCKD: Low-calorie ketogenic diet
MAD: Modified Atkins Diet
VLCnKD: Very low calorie not ketogenic diet
LCD: Low carbohydrate diet
FM: Fat Mass
FFM: Fat-Free Mass
BMI: Body Mass Index
MIDAS: Migraine Disability Assessment Test
BIA: Bioelectrical Impedance Analysis
HIT-6: Headache Impact Test 6
ICW: Intracellular Water
ECW: Extracellular Water
SD: Standard deviation
OR: Odds ratio
CI: Confidence interval
NRS: Numeric Pain Rating Scale
MDD: Major depressive Disorder
MOH: Medication overuse headache
CGRP mABs: Calcitonin Gene-Related monoclonal antibodies
